# Comparative 3D genome analysis between neural retina and retinal pigment epithelium reveals differential *cis*-regulatory interactions at retinal disease loci

**DOI:** 10.1186/s13059-024-03250-6

**Published:** 2024-05-17

**Authors:** Eva D’haene, Víctor López-Soriano, Pedro Manuel Martínez-García, Soraya Kalayanamontri, Alfredo Dueñas Rey, Ana Sousa-Ortega, Silvia Naranjo, Stijn Van de Sompele, Lies Vantomme, Quinten Mahieu, Sarah Vergult, Ana Neto, José Luis Gómez-Skarmeta, Juan Ramón Martínez-Morales, Miriam Bauwens, Juan Jesús Tena, Elfride De Baere

**Affiliations:** 1 Department of Biomolecular Medicine, Ghent University, Ghent, Belgium; 2 Center for Medical Genetics, Ghent University Hospital, Ghent, Belgium; 3 Centro Andaluz de Biología del Desarrollo, Consejo Superior de Investigaciones Científicas and Universidad Pablo de Olavide, Sevilla, Spain

**Keywords:** 3D genome structure, Hi-C, HiChIP, UMI-4C, Neural retina, Retinal pigment epithelium (RPE), Inherited retinal disease (IRD), *ABCA4*, *Cis*-regulatory element (CRE), Enhancer assay

## Abstract

**Background:**

Vision depends on the interplay between photoreceptor cells of the neural retina and the underlying retinal pigment epithelium (RPE). Most genes involved in inherited retinal diseases display specific spatiotemporal expression within these interconnected retinal components through the local recruitment of *cis*-regulatory elements (CREs) in 3D nuclear space.

**Results:**

To understand the role of differential chromatin architecture in establishing tissue-specific expression at inherited retinal disease loci, we mapped genome-wide chromatin interactions using in situ Hi-C and H3K4me3 HiChIP on neural retina and RPE/choroid from human adult donor eyes. We observed chromatin looping between active promoters and 32,425 and 8060 candidate CREs in the neural retina and RPE/choroid, respectively. A comparative 3D genome analysis between these two retinal tissues revealed that 56% of 290 known inherited retinal disease genes were marked by differential chromatin interactions. One of these was *ABCA4*, which is implicated in the most common autosomal recessive inherited retinal disease. We zoomed in on retina- and RPE-specific *cis*-regulatory interactions at the *ABCA4* locus using high-resolution UMI-4C. Integration with bulk and single-cell epigenomic datasets and in vivo enhancer assays in zebrafish revealed tissue-specific CREs interacting with *ABCA4*.

**Conclusions:**

Through comparative 3D genome mapping, based on genome-wide, promoter-centric, and locus-specific assays of human neural retina and RPE, we have shown that gene regulation at key inherited retinal disease loci is likely mediated by tissue-specific chromatin interactions. These findings do not only provide insight into tissue-specific regulatory landscapes at retinal disease loci, but also delineate the search space for non-coding genomic variation underlying unsolved inherited retinal diseases.

**Graphical Abstract:**

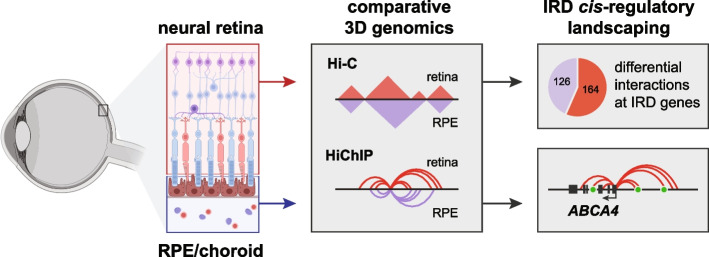

**Supplementary Information:**

The online version contains supplementary material available at 10.1186/s13059-024-03250-6.

## Background

The human retina, the light-sensitive layer of the eye that transmits visual information to the brain, is a highly organized tissue, consisting of a multi-layered neural retina intimately associated with a single layer of retinal pigment epithelium (RPE) and bordered by the choroid, the vascular layer containing blood vessels and connective tissue. Although it is the neural retina that contains the light-sensitive photoreceptor cells, the neural retina as well as the RPE are commonly affected in retinal disease, as the latter plays a crucial role in photoreceptor maintenance and survival [1, 2]. Despite the interconnectedness between these retinal components, they are phenotypically, functionally, and molecularly highly distinct. To illustrate the latter, most known retinal disease genes display a cell-type-specific expression pattern, with large groups being specifically expressed in either photoreceptors or the RPE [3]. 

This type of tissue- or cell-type-specific gene expression is achieved through a tight transcriptional control via thousands of *cis*-regulatory elements (CREs) [4, 5]. Integrated epigenomic analyses have revealed over 50,000 candidate CREs (cCREs) active in the human adult neural retina or RPE, with the majority displaying tissue-specific accessibility [4]. Yet, until recently, linking these cCREs to their true retinal target genes was hampered by the lack of relevant tissue-specific chromatin interaction data. Indeed, spatiotemporal communication between CREs and target promoters relies on a chromatin looping mechanism, ensuring close physical proximity in the three-dimensional (3D) nuclear space [6, 7]. These 3D chromatin interactions are mostly constrained within self-interacting domains, called topologically associating domains (TADs), which are flanked by insulating boundaries enriched for CTCF binding [8]. Although TADs are thought to be largely conserved across cell lines and tissues [8, 9], there have been examples of cell-type specific 3D structures within complex tissues such as the brain [10, 11]. Although a 3D genome map of the human neural retina recently increased our insight into the genetic control of tissue-specific functions [12], 3D genome structure in the RPE/choroid has not been mapped before, nor has it been explored whether differential chromatin interactions exist within the different components of the retina.

Genetic variation disrupting active CREs and/or 3D genome architecture has been reported in inherited retinal disease (IRD), a group of disorders leading to vision impairment and affecting 2 million people worldwide [13, 14]. For instance, duplications within the *PRDM13* and *IRX1* loci, altering enhancer regions, have been associated with North Carolina Macular Dystrophy (NCMD) (MIM #136550 and MIM #608850), a retinal enhanceropathy affecting macular development [15]. Structural variants spanning *YPEL2*, associated with retinitis pigmentosa 17 (RP17) (MIM #600852), have been shown to induce the formation of new TADs (neo-TADs), resulting in ectopic expression of *GDPD1* in photoreceptor cells [16]. So far only a handful of non-coding sequence variants with a regulatory effect have been reported in IRD, as exemplified by single nucleotide variants (SNVs) in two hotspot regions near *PRDM13* [15]. Yet, the highest number of non-coding sequence variants reported in IRD were identified within the *ABCA4* locus, implicated in *ABCA4*-associated IRD (*ABCA4*-IRD, MIM #248200) [17, 18]. Although most of these non-coding variants influence *cis*-acting splicing [17, 19], functional CREs within the *ABCA4* locus may represent targets for hidden genetic variation in *ABCA4*-IRD.

The annotation of functional CREs remains challenging, however, considering the tissue and cell-type specificity of gene regulatory mechanisms. Combining chromatin interaction profiling using C-technologies (e.g., Hi-C, 4C) with epigenomic chromatin signatures generated on relevant human tissues represents a powerful approach to identify cCREs that can be associated with a target gene [20]. Given the increased implementation of whole genome sequencing in genetic testing protocols of rare diseases including IRD [21, 22], prioritizing and identifying key functional regions without coding potential could aid in pinpointing and interpreting overlooked variation associated with disease [23].

Considering the tissue-specificity of gene expression [3] and chromatin accessibility [4] in the two major components of the human retina, we aimed to understand the role of differential 3D chromatin interactions in establishing tissue-specific expression patterns at IRD loci in the human neural retina and the RPE. We therefore generated genome-wide chromatin interaction maps by applying in situ Hi-C [9] and H3K4me3 HiChIP [24] to the neural retina and RPE/choroid from human adult post-mortem donor eyes and performed a comparative 3D genome analysis between these two retinal tissues. We focused in particular on the impact of tissue-specific chromatin interactions at IRD loci and investigated this in depth for the *ABCA4* gene, implicated in the most common autosomal recessive IRD and expressed in both retinal components [3, 4, 25]. Using high-resolution targeted assays (UMI-4C [26]), (single-cell) epigenomic data integration, and in vivo enhancer assays, we characterized tissue-specific *ABCA4* CREs.

## Results

### Comparative 3D genome analysis between the neural retina and RPE/choroid reveals differential interactions

**Fig. 1:**
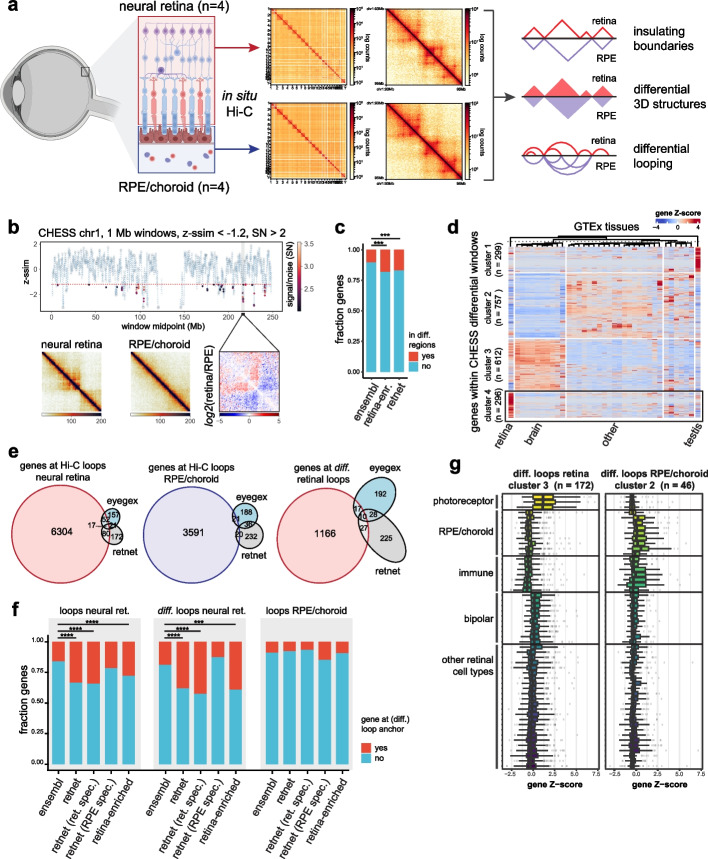
Comparative Hi-C analysis between human neural retina and RPE/choroid. **a** Generation of tissue-specific 3D contact matrices using in situ Hi-C on adult human donor neural retina and RPE/choroid samples (*n* = 4) and strategy for comparative 3D genome analysis. **b** Results of CHESS comparative analysis between the neural retina and RPE/choroid Hi-C contact matrices (z-ssim similarity scores obtained for chromosome 1 using 1-Mb window sizes, z-ssim <  − 1.2, signal/noise (SN) > 2). **c** Enrichment of retina-enriched genes from the EyeGEx database and RetNet IRD genes compared to Ensembl genes within CHESS differential regions (Fisher’s exact test, *p* = 0.000273 and *p* = 0.000658 respectively). **d** Clustered heatmap of genes within CHESS differential windows using GTEx tissue expression data. **e** Overlap between genes at (differential) Hi-C loop anchors identified in neural retina and RPE/choroid and EyeGEx retina-enriched genes and RetNet IRD genes. **f** Enrichment of RetNet IRD genes, retina-specific IRD genes, RPE/choroid-specific IRD genes, and retina-enriched genes from the EyeGEx database compared to Ensembl genes at Hi-C loops in neural retina (Fisher’s exact test, *p* = 4.312e − 13, *p* = 4.875e − 12, *p* = 0.2024 and *p* = 0.0001 respectively), differential Hi-C loops in the neural retina (Fisher’s exact test, *p* = 1.149e − 05, *p* = 1.254e − 06, *p* = 0.7515 and *p* = 3.826e − 13 respectively) and Hi-C loops in RPE/choroid (Fisher’s exact test, *p* = 0.4705, *p* = 0.2559, *p* = 0.0991 and *p* = 0.8237 respectively). **g** Single-cell RNA expression within adult human retina of clusters of genes identified at differential loops in neural retina and RPE/choroid. The figure in panel **a** was partly created using BioRender

As many known retinal disease genes are expressed within specific components and cell types within the human retina [3], we wanted to explore the role of tissue-specific 3D genomic structures or interactions in establishing these expression patterns in the neural retina and RPE/choroid. We used in situ Hi-C on post-mortem human donor retina to map 3D genomic interactions in the adult neural retina (*n* = 4, four eyes from three donors), as well as the RPE/choroid layer (*n* = 4, four eyes from three donors) (Fig. 1a). A total of 1.13 billion and 1.34 billion pairwise genomic contacts could be identified in the neural retina and RPE/choroid, respectively.

These retinal Hi-C maps were subsequently used to calculate genome-wide diamond insulation scores and determine tissue-specific insulating TAD boundaries (Additional file 1: Fig. S1a–c, f–h). We identified 3905 and 3785 boundaries in the neural retina and RPE/choroid respectively, with 60–62% of them overlapping or adjacent in both tissues (Additional file 1: Fig. S1k, Additional files 2 and 3). As expected, these boundaries were enriched for CTCF binding and displayed a convergent orientation bias for CTCF motifs (Additional file 1: Fig. S1d–e, i–j).

Next, we performed a comparative analysis of neural retina vs. RPE/choroid 3D genomes (Fig. 1a). First, we applied the feature-independent CHESS algorithm [27] with both 1 Mb and 500 kb sliding windows to scan the whole genome for quantitative contact differences within the neural retina and RPE/choroid Hi-C maps (Fig. 1b, Additional file 1: Fig. S2–3). Upon merging and reducing overlapping differential windows, we delineated 476 genomic regions displaying differential chromatin interactions (Additional file 4). We identified 2034 protein-coding genes within these differential loci and found, despite the large window sizes used for CHESS analysis, that these were significantly enriched for genes with a highly specific expression in the retina (44/242 retina-enriched genes from the EyeGEx database compared to other GTEx tissues, Fisher’s exact test, *p* = 0.000273) and known IRD disease genes (49/290 RetNet genes, Fisher’s exact test, *p* = 0.000658) (Fig. 1c). Also, by analyzing GTEx RNA expression data for genes within differential regions, we identified a subcluster of 296 genes with highly specific expression in the retina and associated with functions such as “visual perception” (Fig. 1d and Additional file 1: Fig. S4).

As a second approach to determine tissue-specific interactions, we used the retinal Hi-C maps to determine (differential) chromatin looping in neural retina vs. RPE/choroid. Using HICCUPS [9] loop calling, 6884 and 2902 chromatin loops were identified in, respectively, neural retina and RPE/choroid (Additional files 5 and 6). 60% of neural retina loops (4081/6884) correspond to loops previously identified in the same tissue by Marchal et al. [12] Differential loop calling between the neural retina and RPE/choroid resulted in 1292 differential loops, of which 1149 were gained in neural retina and 143 in RPE/choroid (Additional files 7 and 8). We identified all genes with transcription start sites (TSSs) within 2 kb of (differential) loop anchors and found an enrichment of retina-enriched genes and known IRD genes at loops in the neural retina (69/242 retina-enriched genes, Fisher’s exact test, *p* = 2.097e − 06 and 97/290 RetNet genes, Fisher’s exact test, *p* = 4.312e − 13), and at differential loops gained in the neural retina (27/69 retina-enriched genes at retinal loops, Fisher’s exact test, *p* = 0.0001444 and 37/97 RetNet genes at retinal loops, Fisher’s exact test, *p* = 1.149e–05) (Fig. 1e–f). Next, we evaluated whether IRD genes with specific expression in cell types of the neural retina or RPE/choroid would be more strongly associated with tissue-specific loops. Using scRNA-seq data from adult human retina [3] and by scaling gene expression across all identified cell types (Methods), 74/290 IRD genes (RetNet) were identified as having enriched expression in at least one cell type within the RPE/choroid (*Z*-score > 2), while 239/290 IRD genes showed enriched expression in at least one cell type of the neural retina (*Z*-score > 2) (Additional file 1: Fig. S5, Additional file 9: Table S1). We found that retina-specific IRD genes were strongly enriched at (differential) Hi-C loops in the neural retina, while RPE/choroid-specific IRD genes were not enriched at these retinal loops (Fig. 1f). Similarly, at Hi-C loops identified in the RPE/choroid, we observed a 1.7-fold enrichment of only RPE/choroid-specific IRD genes, although this was not significant due to the small number of RPE/choroid loops and therefore small gene sets (Fig. 1f). Gene Ontology enrichment analysis also indicated an involvement of genes associated with the visual system in chromatin looping in the neural retina (Additional file 1: Fig. S6), while enriched terms for genes contacted by RPE/choroid loops included epithelium-associated processes (Additional file 1: Fig. S6). Genes contacted by differential loops in the neural retina showed increased expression in the retina compared to other tissues in the GTEx dataset, while genes at RPE/choroid-specific loops were markedly downregulated in the retina (Additional file 1: Fig. S7–9). Clustering based on tissue-specific expression and subsequent analysis of retinal scRNA-seq data revealed that subsets of genes at differential chromatin loops, displayed specific expression in the most abundant cell types of either the neural retina (photoreceptors) or the RPE/choroid (RPE, fibroblasts, endothelial and immune cells) (Fig. 1g and Additional file 1: Fig. S8, 9).

Taken together, the results from our comparative Hi-C analysis suggest that tissue-specific 3D interactions exist within the adult human retina and could contribute to tissue-specific regulation of genes, including known IRD genes and genes specifically expressed in the retina.

### Mapping *cis*-regulatory retinal landscapes at high resolution using HiChIP

While Hi-C interaction maps provided a genome-wide view of 3D genome architecture in the human retina and RPE/choroid, the sensitivity to identify chromatin loops at high resolution was limited. To identify *cis*-regulatory interactions involving active promoters at a higher resolution and with greater sensitivity, we performed HiChIP [24] for H3K4me3 in both human adult neural retina (*n* = 2, two eyes from one donor) and RPE/choroid (*n* = 2, two eyes from two donors). Visual inspection of HiChIP contact matrices at 5 kb resolution revealed promoter-centered interactions in the form of discrete lines that delineate regulatory landscapes of active genes and were not detectable in the Hi-C heatmaps (Additional file 1: Fig. S10a). Moreover, our HiChIP-derived ChIP-seq signals recapitulated publicly available H3K4me3 datasets (Marchal et al. [12], ENCODE) (Additional file 1: Fig. S10b) and showed the expected enrichment at peaks (Additional file 1: Fig. S10c). Furthermore, we found a high degree of overlap between HiChIP loops and Hi-C loops involving TSSs with invariant H3K4me3. Respectively 72% and 60% of retinal and RPE/choroid Hi-C loops were also present in corresponding HiChIP loops sets, while 75% of retinal Hi-C loops previously identified by Marchal et al. [12] correspond to neural retina HiChIP loops (Additional file 1: Fig. S10d). Yet, distances between anchors of HiChIP loops were significantly smaller (*p*-value < 2.2e − 16, Wilcoxon rank-sum test; Additional file 1: Fig. S10e), the median distance being ~ 115 kb compared with ~ 250 kb of Hi-C loops. We further observed that only a small proportion of HiChIP loops cross TAD boundaries (10.7% and 3% in neural retina and RPE/choroid, respectively, compared to ~ 16% and ~ 13% in shuffled boundary controls; Additional file 1: Fig. S10f), in agreement with preferential intra-domain promoter-enhancer contacts provided by TAD insulation [8, 9, 28].

**Fig. 2:**
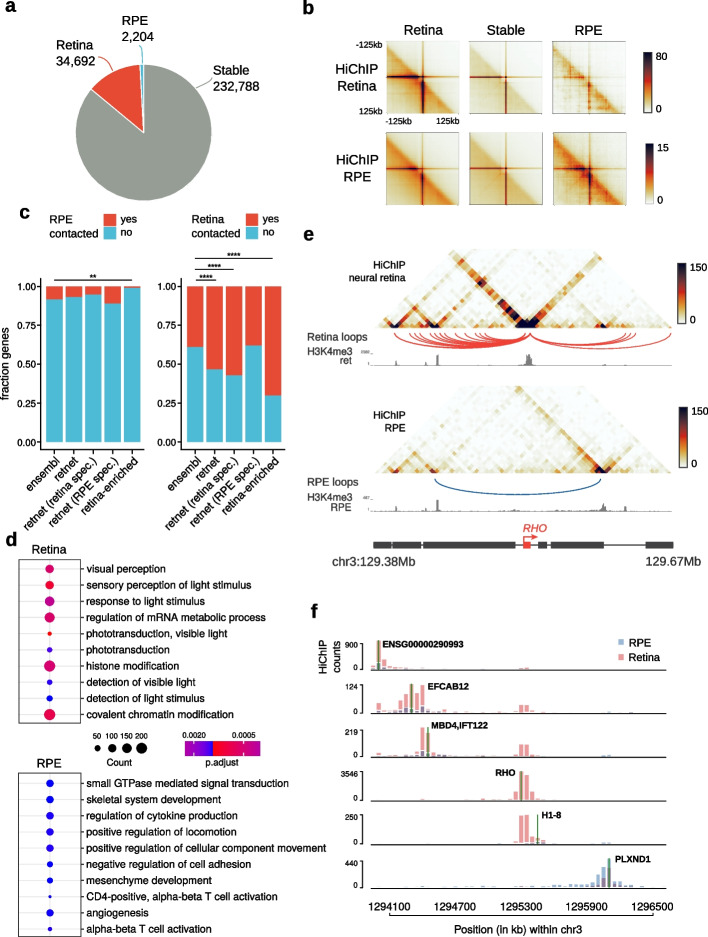
Differential promoter looping between human neural retina and RPE/choroid. **a** Proportion of differential promoter-associated loops (at 5-kb resolution) in human neural retina (red) and RPE/choroid (blue) according to FitHiChIP (FDR < 0.05). **b** Aggregate peak analysis centered at HiChIP loops specific of neural retina, RPE/choroid, and stable loops. **c** Enrichment of RetNet IRD genes, retina-specific RetNet genes, RPE/choroid-specific RetNet genes, and retina-enriched genes from the EyeGEx database within genes specifically contacted in the neural retina (right; Fisher’s exact test, *p* = 7.713e − 06, *p* = 2.042e − 07, *p* = 0.8979, and *p* = 4.382e − 10, respectively) and RPE/choroid (left; Fisher’s exact test, *p* = 0.4856, *p* = 0.1584, *p* = 0.3647, and *p* = 0.0046, respectively). **d** Top-10 enriched GO Biological Process terms associated with differentially HiChIP-contacted promoters in neural retina and RPE/choroid. **e** Genomic tracks showing the 3D chromatin configuration of the *RHO* gene locus. For both tissues, HiChIP contact matrices, differential loops, and HiChIP-derived H3K4me3 ChIP-seq signals are represented from top to bottom. **f** Virtual 4C contact frequencies (viewpoints indicated by a green line) for all genes within the *RHO* locus derived from the neural retina and RPE/choroid binned HiChIP counts

To identify specific HiChIP contacts of both retinal compartments, we performed differential loop calling using FitHiChIP [29]. To unambiguously assign differential contacts due to changes in 3D structure, only interactions with similar ChIP-seq coverage of H3K4me3 in both tissues were considered. We identified 269,684 loops contacting 16,648 promoters that fulfilled this condition, from which 34,692 (from 6463 genes) and 2204 loops (from 1339 genes) were specific of neural retina and RPE/choroid, respectively (Fig. 2a, Additional file 10), in line with the unbalanced difference observed in our Hi-C datasets. Differential intensities were confirmed by aggregate peak analysis plots (Fig. 2b). At retina-specific loops, we found an enrichment in known IRD disease genes and retina-enriched genes from the EyeGEx database (133/249 RetNet genes at retinal HiChIP loops, Fisher’s exact test, *p* = 7.713e − 06 and 71/101 retina-enriched genes at retinal HiChIP loops, Fisher’s exact test, *p* = 4.382e − 10) (Fig. 2c). Moreover, we again found a stronger enrichment when only considering IRD genes with specific expression in cell types of the neural retina (119/208 retina-specific IRD genes at retinal HiChIP loops, Fisher’s exact test, *p* = 2.042e − 07), while RPE/choroid-specific IRD genes were not enriched at retina-specific HiChIP loops (Fig. 2c). Conversely, only RPE/choroid-specific IRD genes were slightly enriched (1.3-fold) at RPE/choroid-specific HiChIP loops (not significant), while we also observed a significant depletion of retina-enriched genes from the EyeGEx database (Fisher’s exact test, *p* = 0.0046) (Fig. 2c).

Examples of RetNet genes associated with tissue-specific contact gains included *ACO2*, *CRX*, *RHO*, *NRL*, and *PROM1* (gain in the neural retina), as well as *CDH3* and *TIMP3* (gain in RPE/choroid) (Additional file 1: Fig. S11). Gene Ontology analysis further revealed enriched biological processes associated with light perception for genes specifically contacted in retina, while RPE/choroid-contacted genes were involved in extracellular matrix organization (Fig. 2d). Additionally, the analysis of GTEx tissue expression data and scRNA-seq data for adult human retina indicated a large cluster of 700 + retina-specific genes involved in retina-specific looping, which were primarily expressed in photoreceptors (Additional file 1: Fig. S12). Expression of genes at RPE/choroid-specific loops was detected across many human tissues, with single-cell data confirming expression of these genes in cell types of the RPE/choroid (Additional file 1: Fig. S13). This was in line with expectations, as the cell types found within the RPE/choroid are also present in epithelial, connective, and vascular tissues throughout the human body, while the retinal tissue from the EyeGEx database primarily contains neural retina [30].

Next, we used these stable and retina-/RPE-specific loops to identify interactions between promoters and candidate *cis*-regulatory elements (cCREs) with activity in the retina or RPE previously identified by Cherry et al. [4] (Additional file 10). Specifically, using HiChIP stable, retina-specific, and RPE-specific loops, we identified 134,374 neural retina loops (stable and retina-specific) connecting 15,819 TSSs to 32,425 retinal cCREs; and 118,461 loops in RPE/choroid (stable and RPE/choroid-specific) connecting 13,190 TSSs to 8060 RPE cCREs.

Illustrative of the power of HiChIP to delineate tissue-specific *cis*-regulatory landscapes was the differential 3D wiring we observed at the *RHO* locus, where neighboring genes formed mutually exclusive contacts in either retinal compartment (Fig. 2e). To further inspect changes in chromatin 3D interactions within this locus, we generated virtual 4C contacts from the HiChIP data for every gene promoter in this region. As inferred from the HiChIP heatmaps, *RHO*/*H1-8* and *PLXND1* genes showed little contact overlap, with most of their interactions mapping to opposing sides of the locus (Fig. 2f).

Altogether, these HiChIP data support the outcome of our comparative Hi-C analysis and extend these results by including high-resolution promoter interactions. This enabled us to refine tissue-specific maps of *cis*-regulatory landscapes in the adult retina and should aid in unraveling the regulatory mechanisms governing retinal disease genes.

### Differential 3D topology and *cis*-regulatory interactions shape IRD loci

**Fig. 3:**
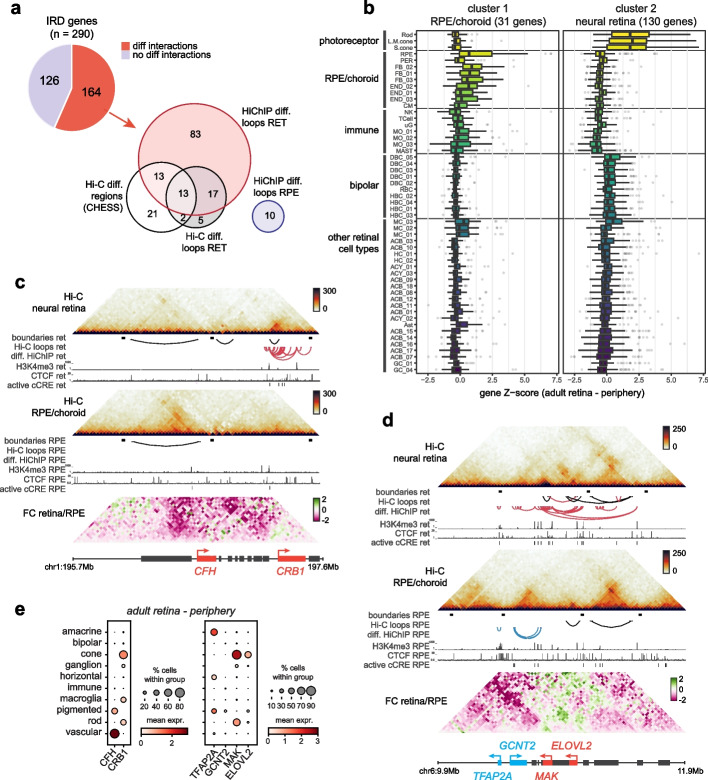
The impact of differential 3D genomic interactions at retinal disease loci. **a** Number of inherited retinal disease (IRD) genes associated with differential interactions in neural retina vs. RPE/choroid through Hi-C differential regions (CHESS) or loops and HiChIP differential loops. **b** Single-cell RNA expression per cell type within the adult human retina of two clusters of IRD genes associated with differential interactions. Cell types: rod, L/M cone, S cone, retinal pigment epithelium (RPE), pericyte (PER), fibroblast (FB), endothelial (END), melanocyte (CM), T-cell, microglia (uG), monocyte (MO), mast cell (MAST), ON bipolar (DBC), rod bipolar (RBC), OFF bipolar (HBC), Müller cell (MC), GABA amacrine (ACB), horizontal cell (HC), GLY amacrine (ACY), astrocyte (AST), ganglion cell (GC). **c** Differential 3D interactions at the *CFH* and *CRB1* locus. **d** Differential 3D interactions at the *MAK* locus. **e** Single-cell RNA expression of genes within highlighted loci in adult human retina (periphery) averaged per cell type group

As single-cell RNA sequencing experiments have indicated that many known IRD genes are expressed in a cell-type-specific manner [3], we used our differential Hi-C and HiChIP interaction data to explore whether tissue-specific interactions at IRD loci could be associated with their specific expression patterns. Considering results from both the Hi-C and HiChIP comparative analyses, 56% of IRD genes (164/290) could be associated with differential 3D interactions (Fig. 3a, Additional file 9: S1). Based on their cell-type-specific expression pattern (single-cell expression data was available for 161/164 genes [3]), we observed two clusters within this subset of IRD genes marked by tissue-specific 3D topology, with the largest cluster predominantly composed of IRD genes specifically expressed in rod and cone photoreceptors, the most abundant cell types in the neural retina, and a small cluster of genes expressed in the RPE or choroidal cell types, including vascular cells, immune cells and fibroblasts (Fig. 3b, Additional file 1: Fig. S14).

The differential Hi-C and HiChIP analyses primarily enabled the identification of IRD genes associated with interaction gains in the neural retina (Fig. 3a). For many of these loci, including all those identified through the three individual analyses (*CC2D2A*, *CEP164*, *DMD*, *ELOVL4*, *EYS*, *GNB3*, *IMPG1*, *LCA5*, *PCDH15*, *PROM1*, *RPGR*, *SAMD7*, *UNC119)*, we found increased local interactions in the neural retina to be correlated with their specific expression in the same tissue (Additional file 1: Fig. S15). In particular, we often observed tissue-specific chromatin looping between genes with similar expression patterns, indicating these might share a regulatory mechanism. For example, *UNC119* (~ cone-rod dystrophy and maculopathy, MIM #620342) forms a retina-specific loop with the *VTN* gene (specifically expressed in cones in the fovea), *ELOVL4* (~ Stargardt-like disease, MIM #600110) contacts *LCA5* (~ Leber congenital amaurosis, MIM #604537), while *SAMD7* (candidate modifier of IRD [31] and macular dystrophy, MIM #620762) forms retina-specific loops, mediated by retina-specific CTCF binding at the *SAMD7* promoter, with both downstream gene *GPR160* and upstream gene *MYNN* (both expressed in photoreceptors) (Additional file 1: Fig. S15b, d, f). Some IRD loci, such as *CC2D2A*/*PROM1* and *IMPG2*, even showed an increase of long-range, inter-TAD contacts with genes displaying a similar expression profile in the neural retina (Additional file 1: Fig. S15g, k). For other genes, we identified tissue-specific contacts with cCREs. *PCDH15* (~ Usher syndrome, MIM #601067) contacts intronic and upstream cCREs through retina-specific loops, both *IMPG1* (~ macular dystrophy, MIM #616151; RP, MIM #153870) and *EYS* (~ RP, MIM #602772) form retina-specific loops with intronic cCREs mediated by retina-specific CTCF binding, while *RPGR* and *DMD* (from its retinal promoter) engage in retina-specific looping with upstream cCREs (Additional file 1: Fig. S15a, c, h, i, fj).

A smaller subset of IRD genes could be associated with interaction gains in the RPE/choroid. Many of these genes displayed specific expression in the RPE or choroidal cell types and could be identified through differential HiChIP chromatin looping (e.g. *CDH3*, *EFEMP1*, *FBLN5*, *LRAT*, *TIMP3*) or local interaction frequency gains detected through CHESS analysis of the Hi-C data (e.g., *AHR*, *CFH*, *CWC27*, *NR2F1*, *PEX7*, *VCAN*, *WFS1*) (Additional file 1: Fig. S16).

Interestingly, a few loci displayed specific contact gains in both the neural retina and RPE/choroid. For example, we observed increased interaction between the *CFH* promoter (~ age-related macular degeneration, MIM #610698) and its upstream region in the RPE/choroid, coinciding with specific expression and increased CTCF binding in the same tissue, while the opposite is true for the nearby *CRB1* gene, which displayed increased local interactions and expression in the neural retina (Fig. 3c, e). This was also the case for the RP-associated *MAK* locus, which in addition to a retina-specific interaction between the *MAK* and *ELOVL2* genes (both specifically expressed in photoreceptors) also showed an increase of local RPE/choroid-specific interactions at the *GCNT2* and *TFAP2A* genes (both expressed in RPE/choroid) (Fig. 3d, e).

### 3D interactions define the *ABCA4 cis*-regulatory landscape in neural retinal and RPE/choroid

Next, we investigated the 3D topology and *cis*-regulatory landscape of an IRD locus in greater detail. We focused on the *ABCA4* locus, implicated in the most common autosomal recessive IRD. The *ABCA4* gene is mainly expressed in photoreceptor cells within the neural retina [32], but has also been shown to be expressed in the RPE [25]. Interestingly, *ABCA4*-IRD has been hypothesized to originate from a fovea-specific dysfunction of RPE cells [3, 25]. Moreover, its genetic architecture is characterized by a high proportion of non-coding pathogenic variants [17, 18]. The retinal Hi-C and HiChIP maps generated here indicated differential chromatin looping and a TAD boundary shift at the *ABCA4* locus, suggesting that specific interactions with distinct CREs in neural retina vs. RPE/choroid could be involved in the differential transcriptional regulation of *ABCA4* (Additional file 1: Fig. S17).

**Fig. 4:**
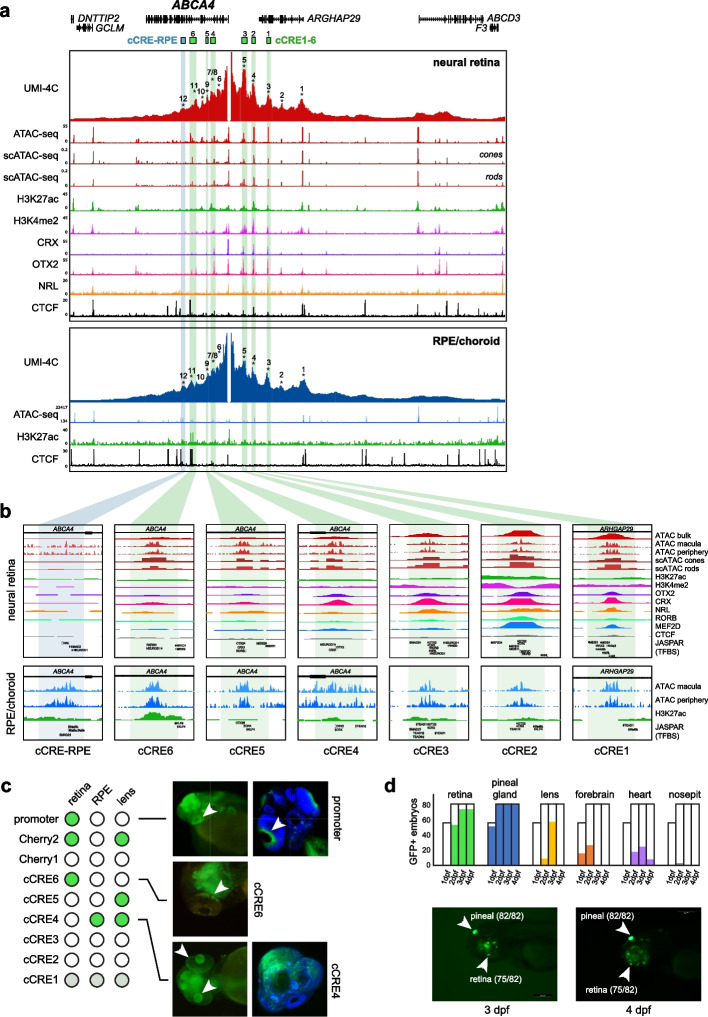
Characterization of the *ABCA4* *cis*-regulatory landscape in human retina. **a** *ABCA4* promoter interaction frequencies using UMI-4C in human neural retina and RPE/choroid from retinal donors (*n* = 3, interacting regions (IRs) indicated 1–12). Candidate *cis*-regulatory elements (cCREs) within IRs were identified using publicly available epigenomic data from human retina: ATAC-seq from bulk retina and scATAC-seq from photoreceptor cells; ChIP-seq for histone marks H3K27ac and H3K4me2, retinal transcription factors (TFs) (CRX, OTX2, and NRL) and the architectural protein CTCF. Epigenomic data for RPE/choroid included bulk ATAC-seq and ChIP-seq targeting H3K27ac and CTCF. All these data were integrated to finely map cCREs. **b** Close-up of the cCREs including the above-described datasets; retinal TF binding (CRX, OTX2, NRL, RORB, and MEF2D); and sequence motifs (Jaspar Core Pred. TFBS 2022) for TFs expressed in photoreceptors (i.e., MEIS1, NRL, NR2E3, OTX2, CRX, MEIS2, MEF2D, RORB, RXRG, SMAD2 and NEUROD1); and the TFs expressed in RPE (CRX, KLF4, KLF9, LHX2, MEIS1, MEIS2, OTX2, RORB, SMAD2, STAT5B, TEAD1, and TEAD3). **c** Overview of in vivo enhancer assays using zebrafish stable transgenic lines; dot plot (left) indicating in which tissues GFP + reporter expression was observed (retina, RPE, and lens, white arrows). **d** Overview of in vivo enhancer assays for the cCRE1–5 synthetic construct through transient transgenesis in zebrafish; bar plots (top) indicating the frequency of GFP + tissues (retina, pineal gland, lens, forebrain, heart, and nosepit) among total GFP + embryos at 1, 2, 3, and 4 days post-fertilization (dpf); example of reporter expression in retina and pineal gland at 3 and 4 dpf

To validate regulatory interactions and to identify interacting cCREs, we performed UMI-4C on human adult neural retina and RPE/choroid using the *ABCA4* promoter and four other viewpoints within the *ABCA4* TAD as bait regions. UMI-4C interaction profiles confirmed extended interactions in the neural retina, as far upstream as the *ABCD3* gene (~ 300 kb), as observed through Hi-C and HiChIP (Additional file 1: Fig. S17, 18). Interactions in the RPE/choroid, on the other hand, appeared to be constrained by a TAD boundary located intergenically between *ARHGAP29* and *ABCD3,* ~ 200 kb upstream of *ABCA4* (Additional file 1: Fig. S17, 18). However, local *ABCA4* interaction frequencies with putative regulatory regions were highly similar (Fig. 4a, Additional file 1: Fig. S19). Within both neural retina and RPE/choroid, we delineated twelve interacting regions (IR1–IR12), five located upstream of the *ABCA4* promoter and seven located within *ABCA4* introns (Fig. 4a, Additional file 9: Table S2). Six of these interactions (IR1, IR4, IR5, IR9, IR11, and IR12) were also confirmed using reverse UMI-4C experiments (Additional file 1: Fig. S18–19). Notably, *ABCA4*-IR12 contacts appeared to be more frequent in the RPE/choroid, while reverse UMI-4C for both IR11 and IR12 revealed a distal RPE-specific interaction spanning ~ 300 kb that was not observed in the neural retina (Additional file 1: Fig. S19). Examination of Hi-C maps of the *ABCA4* locus confirmed that this RPE-specific interaction coincides with the TAD boundaries observed within the RPE/choroid (Additional file 1: Fig. S17).

Subsequently, we identified tissue-specific cCREs within these IRs using publicly available epigenomic datasets (Fig. 4a). Almost all IRs were associated with open chromatin in the neural retina (11/12) [4, 33]; and all of them in RPE (12/12) [33]. In addition, we found histone modifications associated with active enhancers (H3K27ac and H3K4me2) and photoreceptor-specific transcription factors (TFs) (e.g., OTX2, CRX, NRL, RORB, and MEF2D), including their sequence motifs, to be present at most IRs within the neural retina (10/12, Additional file 9: Table S2). Within the RPE, we identified the presence of H3K27ac within 6 of 12 IRs, in addition to the presence of TF sequence motifs found to be expressed in the RPE (e.g., KLF4, LHX2, OTX2, and TEAD1) (Additional file 9: Table S2). Of note, IR12 appears to contain a cCRE with RPE-specific activity given the presence of H3K27ac and high frequency of chromatin accessibility (cCRE-RPE, Fig. 4b), as also reported by Cherry et al. [4].

### Single-cell dissection of the *ABCA4 cis*-regulatory network reveals cCREs in photoreceptors and RPE

Given the cellular complexity of the retina, we mined the *ABCA4* locus in publicly available scATAC-seq and scRNA-seq datasets derived from human neural retina [34]. Using these datasets, we could identify the precise cell type in which cCREs within 9/11 IRs are likely active (Additional file 1: Fig. S20, Additional file 9: Table S2). As expected, we observed the highest frequency of chromatin accessibility at the *ABCA4* TSS among adult rod and cone photoreceptor cells, which correlated with transcriptional activity in these cell types (Additional file 1: Fig. S20a). Also, most IRs (9/11) were found to be accessible in at least one retinal cell cluster and could be linked to the *ABCA4* promoter through co-accessibility analysis, corroborating the UMI-4C interaction profiles (Additional file 1: Fig. S20b, Additional file 9: Table S2). Of all IRs, seven were found to be accessible in photoreceptor cells while only one, the *ARHGAP29* promoter (IR1), was found to be constitutively accessible. Interestingly, IR8 and IR10 were found to be exclusively accessible in the adult Müller glial cells, in which low *ABCA4* expression can be observed (Additional file 1: Fig. S20).

Overall, upon cell-type-specific epigenetic characterization of the IRs and narrowing down to elements active in photoreceptor cells, we prioritized six cCREs (cCRE1-6), within IR3, IR4, IR5, IR7, IR9, and IR11 respectively, as candidate regulatory elements for *ABCA4* expression (Fig. 4b and Additional file 9: Table S2). Moreover, the available TF ChIP-seq data and motifs found in the center of these cCREs suggest that CRX, OTX2, NRL*,* and RORB likely constitute the core TFs necessary for *ABCA4* transcriptional regulation in photoreceptors cells (Fig. 4b and Additional file 9: Table S2). Note that since some of these TFs are expressed in the RPE as well (CRX and OTX2), the proposed cCREs may also act as *cis*-regulators in this cell type.

### In vivo zebrafish enhancer assays characterize *ABCA4* cCRE activity

To further evaluate the activity pattern of cCREs with a putative role in *ABCA4* regulation, in vivo enhancer assays in zebrafish were performed. We prioritized eight elements for functional assessment, including the *ABCA4* promoter, five out of the six cCREs (cCRE2-6) identified above, as well as two previously identified cCREs by Cherry et al. [4] that had not been tested in vivo before (Cherry1/2) (Fig. 4c, Additional file 9: Table S3) [9]. In total, we generated eight stable transgenic zebrafish lines and assessed GFP fluorescence at 1, 2, and 3 days post fertilization (dpf) to evaluate enhancer activity. Reporter expression in the eye was observed for the majority of the tested elements (5/8) (Fig. 4c, Additional file 1: Fig. S21). From these, three exhibited reporter expression in the retina (promoter, cCRE6, Cherry2), three in the lens (cCRE4, cCRE5, and Cherry2), and one in the RPE (cCRE4) (Fig. 4c, Additional file 1: Fig. S21).

To assess whether cooperativity between several cCREs could improve tissue-specificity, we designed a synthetic construct including core elements of 5 out of the 6 prioritized cCREs (cCRE1–5), since ChIP-seq data [4] indicated these were bound by a common set of photoreceptor TFs (CRX, NRL, OTX2, RORB, and MEF2D) (Additional file 9: Table S2). This construct was cloned into the E1b-tol2 vector [35] and transient eGFP expression was annotated at one, two, three, and four dpf. Remarkably, we observed robust and strong reporter expression in the retina (75/82) and pineal gland (82/82) (Fig. 4d, Additional file 1: Fig. S22, Additional file 9: Table S4). Of note, the pineal gland contains both rod and cone light-sensitive photoreceptor cells and plays important roles in the regulation of circadian rhythms in animal behavior and physiology [36]. Overall, these results indicate a functional role of the proposed cCREs and suggest a mechanism of enhancer cooperativity to ensure tissue-specific *ABCA4* expression.

## Discussion

Through extensive 3D genome mapping, including genome-wide (Hi-C), promoter-centric (HiChIP), and locus-specific (UMI-4C) profiling, we have characterized the 3D chromatin architecture and *cis*-regulatory interactions in the two major components of the human retina, the neural retina, and the RPE/choroid. A comparative analysis between these two tightly interconnected layers revealed differential 3D chromatin topology and *cis*-regulatory interactions at loci associated with tissue- and cell-type specific expression and/or retinal disease. Importantly, we found that almost 60% of known IRD genes were marked by a differential 3D genome topology.

Recently Marchal et al. [12] mapped high-resolution 3D topology of the human retina by Hi-C, and by integrating this with chromatin accessibility, histone marks, and transcriptome data of the human retina provided insight into targets of CREs and into the chromatin architecture of super-enhancers. Here, combining two complementary genome-wide chromatin interaction profiling technologies, in situ Hi-C and H3K4me3 HiChIP, allowed us to investigate multiple aspects of differential 3D topology in the neural retina vs. RPE/choroid. The comparative Hi-C analyses provided a genome-wide view on interaction frequency changes, primarily revealing increased *cis*-regulatory interactions near genes displaying specific expression in the most abundant cell types of either the neural retina (*i.e.* rod and cone photoreceptors) or the RPE/choroid. These interactions appeared to facilitate contact with tissue-specific cCREs or other genes with similar expression profiles. The inclusion of HiChIP analyses greatly increased the sensitivity with which we could detect differential chromatin looping at active promoters. We therefore focused the differential HiChIP analysis on genes that were active in both retinal compartments, revealing differential usage of cCREs for gene regulation in both tissues.

The 3D interaction differences between the two closely related tissues highlighted in this study stress the importance of acquiring tissue-specific interaction data for genes with highly specific expression patterns, as is the case for most retinal disease genes. This type of tissue-specific data is crucial to correctly interpret *cis*-regulatory landscapes and disease-associated variation, in particular within the non-coding genome. Yet, it is important to note that even chromatin interaction mapping at the tissue level foregoes the underlying cellular complexity, as the resulting interaction maps reflect contact frequencies derived from a mixture of different cell types. In this case, we observed that interaction data from the neural retina primarily reflects contacts derived from the most abundant cell types by far, namely the photoreceptors. This was clearly exemplified by the photoreceptor-specific expression of most genes near differential contacts gained in the neural retina maps. The RPE/choroid layer, on the other hand, is comprised of a mixture of epithelial, endothelial, fibroblast, and immune cells, and the resulting interaction maps are therefore expected to reflect an average contact frequency across these different cell types. This might also explain the imbalance we observed in the number of chromatin loops that could be identified in neural retina vs. RPE/choroid Hi-C matrices. Despite similar sequencing coverage and contact numbers, more than twice as many Hi-C loops were identified in the neural retina. We speculate that the punctate signal from cell-type specific loops might be diluted in the RPE/choroid interaction maps due to its heterogeneous composition. An alternative explanation though may come from a lower degree of *cis*-regulatory complexity in the RPE/choroid compartment versus neural retina, given that neurons in general are highly complex cell types from a regulatory point of view [37]. Future interaction mapping at the cell-type level will be required to disentangle this complexity.

To investigate the potential impact of differential 3D chromatin architecture on IRD genes in greater detail, we focused on the *ABCA4* locus, which was marked by a shift in TAD boundaries, as well as differential chromatin looping in our comparative analysis. Cherry et al. [4] previously annotated cCREs of the *ABCA4* region in the human retina, based on tissue-specific epigenomic markers, TF binding, and gene expression datasets. Here, integration of chromatin conformation, scATAC-seq, and scRNA-seq datasets revealed six cCREs interacting with *ABCA4* and presumably active in photoreceptors. These were located “proximally” (~ 75 kb from the TSS), upstream of the promoter, and within intronic regions, as is expected for tissue-specific enhancers [38, 39]. Overall, contact frequencies between the *ABCA4* promoter and these proximal cCREs were highly similar in neural retina and RPE/choroid, except one interaction in the RPE/choroid that contained RPE-specific enhancer marks (cCRE-RPE). To functionally validate these cCREs, zebrafish transgenic enhancer assays were performed using stable lines, revealing expression in relevant tissues such as the retina, lens, and RPE. Since this expression pattern was not specific for photoreceptor cells, we tested the cooperativity of five cCREs and demonstrated specific retinal expression, presumably in photoreceptors. The latter emphasizes the importance of the 3D chromatin architecture for the regulation of tissue-specific *ABCA4* expression and of the tissue-specific CREs involved [40].

The number of genetic defects affecting CREs and/or 3D genome architecture reported in Mendelian retinal diseases is slowly emerging [13, 14]. A striking example where 3D genome topology of patient-derived retinal organoids was used to interpret a non-coding structural variant in IRD, was reported only recently [16]. Relating CREs to their target genes is useful to interpret more subtle variants with a regulatory effect, as reported in NCMD, a retinal enhanceropathy [15]. We anticipate that multi-omics analyses of functional non-coding regions within retinal disease loci, as illustrated here for the *ABCA4* locus, will accelerate our understanding of Mendelian retinal diseases.

## Conclusions

In summary, we have shed light on the extent of differential 3D chromatin landscapes in neural retinal and RPE/choroid, the two major components of the human retina. Given the growing interest of non-coding variation both in multifactorial eye diseases implicating the retina such as age-related macular disease and glaucoma, and Mendelian retinal diseases, a differential annotation of the 3D topology of the retinal compartments, and adequate interpretation of different categories of variants is highly needed. For example, TAD boundaries and chromatin loops within the different retinal compartments, as identified in this study, will allow to define biologically relevant search spaces for missing heritability in complex as well as Mendelian retinal diseases such as *ABCA4* retinopathy, one of the most frequent IRDs.

## Methods

### Tissue preparation and nuclei isolation

Post-mortem human neural retina and RPE/choroid mixtures were obtained through the Tissue Bank of Ghent University Hospital and Antwerp University Hospital under ethical approval of the Ethics Committee of Ghent University (2018/1072, B670201837286). Eye globes were provided with a description of time and cause of death, post-mortem circulation time (ranging from 3-18 h), age, and sex (Additional file 9: Table S5). None of the eight donors had a prior known ophthalmological condition.

The eye globes were dissected on ice, followed by extraction of the neural retina and the RPE/choroid. The resulting tissues were resuspended in 1XPBS supplemented with 10% Fetal Bovine Serum. The samples were processed according to Matelot and Noordermeer [41] and cross-linking of nuclei was performed using 2% formaldehyde. Finally, the obtained nuclei were aliquoted per 10 million and snap frozen after supernatant removal. Samples were stored at – 80 °C.

### Generation of Hi-C libraries

Crosslinked nuclei from four neural retinas and four RPE/choroid samples (derived from four eyes obtained from three donors) were used to construct Hi-C libraries, following the in situ Hi-C protocol adopted by the 4D Nucleome consortium [9] with a few adaptations (Additional file 9: Table S5). Briefly, for each replicate ~ 5 million pre-lysed, crosslinked nuclei were digested overnight using 250 U *Dpn*II restriction enzyme (New England Biolabs, R0543L). DNA ends were marked by incorporating biotin-14-dATP (Life Technologies, 19524–016) and ligated for 4 h using 2000 U T4 DNA ligase (New England Biolabs, M0202L). Subsequently, crosslinks were reversed overnight using proteinase K (Qiagen, 19131) and Hi-C template DNA was purified using 1 × AMPure XP beads (Beckman Coulter, A63881) and stored at 4 °C until library preparation. Hi-C template DNA was sheared to a size of 300–500 bp using microTUBE snap-caps (Covaris, 520045) in a Covaris M220 sonicator and MyOne Streptavidin T1 beads (Life Technologies, 65601) were used to pull down biotinylated ligation junctions. Next, samples were split into 5-µg aliquots for sequencing library preparation using the NEBNext Ultra II DNA Library Prep Kit (New England Biolabs, E7645L) and NEBNext Multiplex Oligos (New England Biolabs, E7335L). Amplified libraries were purified and size selected using 0.55 × and 1.2 × AMPure XP beads (Beckman Coulter, A63881). Pooled libraries were sequenced on an Illumina NovaSeq 6000 using 100-bp paired-end reads to a depth of ~ 500 million reads per sample (total coverage neural retina: 1,818,070,845 reads; RPE/choroid: 2,072,463,026 reads).

### Hi-C data analysis

FASTQ files containing raw sequencing data were processed into Hi-C contact matrices containing both raw and normalized counts using the Juicer pipeline (v1.6) [42] with BWA-MEM mapping (v0.7.17) [43] to the hg38 reference genome. Paired contacts from individual replicates were merged to create mega contact matrices for each tissue. Insulating boundaries between self-interacting domains were identified based on diamond insulation score minima. We used cooltools (v0.5.2, 10.5281/zenodo.5214125.) to calculate a genome-wide contact insulation score with 250 kb window size for SCALE normalized mega Hi-C contact matrices (MAPQ > 30) at 25-kb resolution. Insulating boundaries were determined by applying automated “Li” thresholding (from the scikit-image Python package) on boundary strength. Chromatin loops were identified using HiCCUPS [9] (as implemented in Juicer v1.6), using SCALE normalized mega Hi-C contact matrices (MAPQ > 30) at 5, 10 and 25 kb resolution (parameters as used by Rao et al. [9]: -m 512 -r 5000,10000,25000 -k KR -f 0.1,0.1,0.1 -p 4,2,1 -i 7,5,3 -t 0.02,1.5,1.75,2 -d 20000,20000,50000). Differential loops in neural retina *vs.* RPE/choroid were determined using HiCCUPSDiff (as implemented in Juicer v1.6) with the same parameters and input matrices. Differential 3D features in neural retina *vs.* RPE/choroid were identified using the CHESS algorithm [27]. CHESS was run on a per-chromosome basis with SCALE normalized mega Hi-C contact matrices (MAPQ > 30, 25-kb resolution), using sliding windows of 1 Mb and 500 kb with a 100 kb step size. Top differential windows were filtered using z-ssim <  − 1.2 and signal-to-noise > 2 or 2.5 for the 1 Mb and 500 kb window analysis respectively. Filtered differential windows from both analyses were merged and overlapping windows were collapsed to generate a list of differential regions. We used FAN-C [44] to plot Hi-C matrices and fold-change matrices for regions of interest. All downstream analyses are described in a separate section below.

### Generation of HiChIP libraries

HiChIP was performed as previously described [24] using cross-linked nuclei from two neural retina and RPE/choroid samples (derived from two eyes, obtained from one and two donors respectively) (Additional file 9: Table S5). After lysis, digestion was performed using 400-U DpnII (R0543T-NEB) restriction enzyme. Next, digestion efficiency was assessed and incorporation Master Mix (biotin-dATP 0.4 mM/19524016- Thermo Fisher; dNTP-A mix; and DNA Polymerase I, Large (Klenow) Fragment M0210-NEB) was added to fill in the restriction fragments overhangs and mark DNA ends with biotin in rotation during 1 h at 37 °C. Subsequently, ligation master mix was added (10 × NEB T4 DNA ligase buffer with 10-mM ATP B0202-NEB); 10% Triton X-100, BSA (B9000-NEB), T4 DNA ligase (M0202-NEB), and H2OmQ) and incubated at 16 °C in rotation. Sonication was performed keeping the samples on ice using the M220 Focused-ultrasonicator (Covaris) with the following cycling conditions: duty cycle 10%, PIP 75W, 100 cycles/burst, time 5′. This allowed to obtain DNA fragments of around 300 bp in size which were incubated with Dynabeads Protein G (10003D-TermoFisher) and 6.7 µg with anti-H3K4me3 antibody overnight at 4 °C with rotation. Samples were purified using the DNA Clean and Concentrator columns (D4004-Zymo Research). Up to 150 ng was taken into the biotin capture step, performed using Streptavidin C-1 beads (65,002-ThermoFisher). TAGmentation was conducted using the Nextera DNA Library Preparation Kit (FC-121-1030-Illumina) and library amplification was performed using NEBNext® High-Fidelity 2X PCR Master Mix (M0541L-NEB) with Nextera Ad1_noMX and Ad2.X primers. The resulting product was purified with the DNA Clean and Concentrator columns (D4004-Zymo Research).

### HiChIP data analysis

Paired-end reads were aligned to the hg38 reference human genome using the TADbit pipeline [45] with default settings. Briefly, duplicate reads were removed, *Dpn*II restriction fragments were assigned to resulting read pairs, valid interactions were retained by removing unligated and self-ligated events and multiresolution interaction matrices were generated. To create 1D signal bedfiles, equivalent to those of ChIP-seq, dangling end read pairs were used and coverage profiles were generated in bedgraph format using the bedtools genomecov tool. Next, we performed bedgraph to bigwig conversions for visualization purposes using the bedGraphToBigWig tool from UCSC Kent Utils. 1D signal bedgraph files were then used to call peaks either with nucleR [46] or with MACS2 [47] using the no model and extsize 147 parameters and an FDR ≤ 0.05.

FitHiChIP [29] was used to identify “peak-to-all” interactions at 5-kb resolution using HiChIP filtered pairs and peaks derived from dangling ends. Loops were called using a genomic distance between 20 kb and 2 Mb, and coverage bias correction was performed to achieve normalization. FitHiChIP loops with q-values smaller than 0.05 that were common to both replicates and involving promoters were kept for further analyses. For differential loop calling between the neural retina and RPE/chroroid, we used the script "DiffAnalysisHiChIP" from FitHiChIP with FDR and fold-change thresholds of 0.05 and 1.5, respectively. To avoid the identification of differential loops due to changes in ChIP-seq coverage, only differential loops connecting anchors with similar H3K4me3 intensities were kept (i.e., category ND–ND from the FitHiChIP differential loop calling output). Gene annotation of loop anchors was performed as described in the “Downstream analyses of Hi-C and HiChIP data” section below, and only promoter-associated loops were finally retained.

To determine the overlap between Hi-C loops and HiChIP loops identified in both retinal tissues, FitHiChIP [29] was used to annotate Hi-C loops with H3K4me3 at 5-kb resolution. Hi-C loops were then filtered to only retain those loops with characteristics that resemble those of HiChIP loops included in the differential FitHiChIP analysis, i.e., category ND–ND and 2 kb up- or downstream from a TSS. Subsequently, we performed an overlap between (1) filtered retinal Hi-C loops and stable or retina-specific HiChIP loops and (2) filtered RPE Hi-C loops and stable or RPE-specific HiChIP loops. The same approach was used to perform the overlap between retinal Hi-C loops identified by Marchal et al. [12] and the set of stable or retina-specific HiChIP loops.

Virtual 4C tracks of the RHO gene locus were generated from HiChIP interaction matrices. First, virtual 4C baits were determined by overlapping of HiChIP 5 kb bins with gene promoters located within a 265-kb locus around RHO (chr3:129395000–129660000). Then, we extracted all interaction counts from each single bait belonging to such locus.

For the computation of loops crossing the TAD boundaries of Fig_HiChIP_S6, five sets of shuffled TAD boundaries were generated by partitioning the genome into virtual TADs with the same size as experimental ones but randomly positioned within chromosomes.

### Downstream analyses of Hi-C and HiChIP data

Gene sets used for downstream analyses/annotation of Hi-C and HiChIP differential regions, loops, and boundaries, included Ensembl Human genes (GRCh38.p13), filtered for protein-coding, long non-coding RNA and microRNA transcripts, known IRD genes (Additional file 9: Table S1) and retina-enriched genes from the EyeGEx database (defined as genes having a tenfold or higher expression in the retina than in at least 42 of the 53 GTEx (v7) tissues) [30]. For annotation purposes, a 2-kb region up- and downstream of the TSS was considered. Gene Ontology enrichment of genes at (differential) 3D features was performed using the “clusterProfiler” package in R (ontology = Biological Process, Benjamini–Hochberg adjustment, *q*-value < 0.05) [48]. Fisher’s exact test (*p*-value < 0.05) was used to determine enrichment of gene sets of interest at (differential) 3D features.

Tissue-specific expression of genes in differential windows or at differential loops was evaluated using the GTEx dataset (v8) with integrated EyeGEx expression data for retina [30], as is available through The Human Protein Atlas (v23.0, https://www.proteinatlas.org) [49]. Specifically, normalized expression values (normalized transcripts per million (nTPM)) were log2-transformed and converted to gene *Z*-scores. Clustered heatmaps were generated using the ComplexHeatmap package in R [50].

Single-cell RNA-seq data from the human adult peripheral retina was obtained from Cowan et al. [3] Specifically, we converted cell-type level, normalized gene expression values (expression normalized to 10,000 transcript counts per cell type) to cell-type level gene *Z*-scores. Genes with cell-type specific expression in the RPE/choroid where then identified by filtering for genes with a *Z*-score > 2 in at least one cell-type found in the RPE/choroid layer (“RPE,” “PER,” “FB_01,” “FB_02,” “FB_03,” “END_01,” “END_02,” “END_03,” “CM,” “NK,” “TCell,” “MO_01,” “MO_02,” “MO_03,” “MAST”). Similarly, to identify genes with cell-type-specific expression in the neural retina, we filtered for genes with a *Z*-score > 2 in at least one cell-type found in the neural retina (all other cell-types excluding the ones mentioned above). Clustered heatmaps were generated as described above.

### Generation of UMI-4C libraries and data analysis

The generation of the 3C template was performed as previously described [26]. Briefly, around 5 million cross-linked nuclei were digested overnight using 400 U *Dpn*II (NEB). After digestion, ligation was performed overnight using 4000 U of T4 DNA ligase (NEB), followed by the addition of proteinase K (BIOzymTC). The fficiency of digestion and ligation were evaluated via agarose gel electrophoresis. Next, samples were de-crosslinked, followed by purification of samples using AMPure XP beads (Agencourt). Subsequently, 4 µg of the 3C template was sheared on a Covaris M220-focused ultrasonicator to get 300 bp DNA fragments. The UMI-4C sequencing library preparation was obtained using the NEBNext Ultra II Library Prep Kit (NEB). Library amplification was performed by nested PCR. In the first PCR, 100 ng of the library was amplified using an upstream (US) forward primer and a universal reverse primer using the KAPA2G Robust HotStart ReadyMix (Roche). The resulting product was amplified using a downstream (DS) forward primer and the same universal reverse primer. Primer sequences can be found in Additional file 9: Table S6. Libraries were multiplexed in equimolar ratios and sequenced on the Illumina NovaSeq 6000 platform, resulting in 150 bp paired-end reads. These were demultiplexed based on their barcodes and their DS primer using runcutadapt (https://github.com/marcelm/cutadapt). UMI-4C data was processed using the R package umi4cpackage 0.0.0.9000 (https://github.com/tanaylab/umi4cpackage; https://github.com/tanaylab/umi4cpackage/index.html) [26]. Profiles were generated using default parameters, pooling all samples per viewpoint and condition (retina and RPE/choroid), and using a minimum win_cov of 50. All individual samples were interrogated for the *ABCA4* promoter region. Reverse UMI-4C were performed, using at least 2 different biological replicates (2 different human donors).

### Integration of bulk and single-cell transcriptomic and epigenomic datasets from human donor retina

To predict putative CREs for the *ABCA4* locus, an integration of publicly available datasets based on human neural retinal *post-mortem* material was performed. Data from the following experiments was included: ATAC-seq derived from healthy adult donor retinas [4, 33], scATAC-seq from human embryo and adult post-mortem retinas [34], DNase-seq from ENCODE based on fetal retinas [51] and ChIP-seq of histone modifications (H3K27ac and H3K4me2), specific retinal transcription factors (CRX, OTX2, NRL, CREB, RORB and MEF2D) and CTCF derived from post-mortem donors with no eye condition [4]. Equally, bulk ATAC-seq [4, 33] and ChIP-seq data for the active enhancer marker H3K27ac [4, 33] derived from healthy post-mortem donors were also integrated. A ChIP-seq dataset targeting the CTCF protein derived from primary RPE from ENCODE (ENCSR000DVI) was also included. Additionally, single-nucleus ATAC-seq data [34] of embryonic (53, 59, 74, 78, 113, and 132 days) and adult (25, 50, and 54 years old) human retinal cells were obtained from GSE183684 and imported into R (v4.0.5). The matrices were processed using the ArchR single-cell analysis package (v1.0.1) [52] and processed according to Thomas et al., [34]. After filtering out doublets, the dataset was characterized by 61,313 number of cells. Single-nucleus RNA-seq data [34] for the same tissue types and timepoints were integrated using the unconstrained integration method. Peak calling was performed using the native peak caller “TileMatrix” from ArchR and bigwig files from each annotated cell cluster were extracted and converted to bedgraph files. Peak identification was performed using bdgpeakcall (MACS2.2.7.1) [47] using default parameters and a value of 0.1 as cutoff.

### Generation of in vivo reporter constructs

Eight elements were selected for functional assessment, including the *ABCA4* promoter, five out of the six cCREs (cCRE2–6) prioritized in the study, as well as two previously identified cCREs by Cherry et al. [4] (Cherry1/2) that had not been tested in vivo before. Human genomic DNA (Roche) was amplified, using the Phusion High Fidelity PCR kit (NEB) using primers designed to span the ATAC-seq signals (Additional file 9: Table S6) following the manufacturer’s instructions. PCR products were purified with Isolate II PCR and Gel Kit (BIOLINE) and cloned into the entry vector pCR®8/GW/TOPO (#250020 Invitrogen, ThermoFisher Scientific) according to manufacturer’s instructions. The fragments were then recombined into the destination vector for zebrafish transgenesis using Gateway® LR Clonase® II Enzyme mix (#11791020, Invitrogen, ThermoFisher Scientific), following the manufacturer’s instructions. This vector contains the strong midbrain enhancer z48 and the green fluorescent protein (GFP) reporter gene under the control of the *gata2* minimal promoter [53]. Transformation was performed with MultiShotTM FlexPLate Mach1TM T1R (#C8681201, Invitrogen, ThermoFisher Scientific), grown O.N. at 37 °C. Vector selection was performed with 100 μg/ml Ampicillin (#624619.1, Normon). Plasmids were purified with NZYMiniprep kit (#MB010, NZYTech) and validated using Sanger sequencing. Final plasmids were purified with phenol/chloroform (#A931I500 and #C/4920/15, Fisher Chemical) and concentration was determined using Qubit (Invitrogen).

### Functional characterization of cCREs using in vivo enhancer assays in zebrafish

All zebrafish lines were generated through Tol2-mediated transgenesis [54]. Tol2 cDNA was transcribed by Sp6 RNA polymerase (#EP0131, ThermoFisher Scientific) after Tol2-pCS2FA vector linearization with *Not*I restriction enzyme (#IVGN0016, Anza, Invitrogen, ThermoFisher Scientific). All constructs were microinjected into the yolk of > 200 wild-type zebrafish embryos at the single-cell stage using the Tol2 transposase system for germline integration of the transgene according to Bessa et al. [55] with minor modifications. As a readout, GFP fluorescence was observed and its localization was annotated at 1, 2, and 3 days post fertilization (dpf) to evaluate enhancer activity, using GFP expression in the midbrain as transgenesis control.

As GFP reporter expression becomes masked by the pigmentation of the eye as the RPE develops, embryos were also treated with PTU to decrease eye pigmentation [56].

### Supplementary Information

**Additional file 1.** Supplementary Figures S1-22 (.pdf).

**Additional file 2.** BED file with Hi-C boundaries in neural retina (25 kb resolution).

**Additional file 3.** BED file with Hi-C boundaries in RPE/choroid (25 kb resolution).

**Additional file 4.** BED file with Hi-C CHESS regions with differential 3D topology between neural retina vs. RPE/choroid.

**Additional file 5.** BEDPE file with Hi-C loops in neural retina.

**Additional file 6.** BEDPE file with Hi-C loops in RPE/choroid.

**Additional file 7.** BEDPE file with differential Hi-C loops gained in neural retina.

**Additional file 8.** BEDPE file with differential Hi-C loops gained in RPE/choroid.

**Additional file 9.** Supplementary Tables S1-6 (.xlsx).

**Additional file 10.** BED file with stable and differential HiChIP loops in neural retina and RPE/choroid, with annotation of interacting *cis*-regulatory elements (CREs, from [4]).

**Additional file 11.** Peer review history.

## Data Availability

All datasets generated in this study have been deposited in NCBI’s Gene Expression Omnibus and are accessible through GEO Series accession number GSE236022 (https://www.ncbi.nlm.nih.gov/geo/query/acc.cgi?acc=GSE236022).
