# Supplementary material for: Comparative 3D genome analysis between neural retina and retinal pigment epithelium reveals differential cis-regulatory interactions at retinal disease loci
Source: Genome Biol. 2024 May 17;25:123. doi: 10.1186/s13059-024-03250-6 (PMC11100165; doi:10.1186/s13059-024-03250-6)
Supplement: Supplementary file 11 — Additional file 11. Peer review history. [file 13059_2024_3250_MOESM11_ESM.docx]

**Review history**

**First round of review**

**Reviewer 1**

The manuscript "Comparative 3D genome analysis between neural retina and RPE reveals differential cis-regulatory interactions at retinal disease loci" by D'haene et al., compares for the first time genome-wide chromatin 3D interactions between human neural retina and retinal pigment epithelium (RPE)/choroid (vascular bed in outer layer of retina) using Hi-C and a higher resolution assay, HiChIP. They then investigate how the retina-specific and RPE-specific interaction maps can help identify cis-regulatory effects on 290 inherited retinal degeneration (IRD) disease genes, and find differential chromatin interactions are enriched in 60% of IRD genes. They further describe the biological processes enriched for genes in retina or RPE-specific chromatin contacts. Finally, the authors focus on an IRD gene example, ABCA4 that makes up a significant fraction of early onset retinal degeneration cases and is the most common autosomal recessive IRD gene expressed in both neural retina and RPE. They identify tissue-specific chromatin interactions in ABCA4 in neural retina and RPE, and use a targeted high resolution assay (UMI-4C2), in vivo enhancer assays in zebrafish, and integration with single cell epigenomic data to provide additional support and further characterize the tissue-specific cis regulatory elements in ABCA4. The analyses presented in this paper appropriately support the conclusions of this study.

This work provides an important resource for the study of both rare and common retina-related eye disease genes and variants, and proposes an experimental and computational workflow that can be applied to other tissue and disease systems. The manuscript is well written. I have a few suggestions and mostly minor comments I recommend addressing:

Major comments:

1) If there are enough IRD genes known to specifically affect the RPE compared to neural retina, it would be interesting to evaluate the concordance of RPE-specific IRD genes with genes enriched in RPE-specific chromatin loops or chromatin interaction with promoters, and IRD genes manifested specifically in the neural retina and the retina-specific chromatin interactions.
2) How do the retina loops found in this study compare to those found in Marchal et al., 2022?
3) The authors define retina-enriched genes from the EyeGEx database (Ratnapriya et al , Nature Genetics 2019) as genes with >10-fold higher expression in retina compared to >=42 of 53 GTEx (v7) tissues. It would be helpful to define what is meant by retina-enriched genes in the Results section, i.e., genes expressed at higher levels in retina compared to GTEx tissues. The authors might want to consider calling these genes "retina-specific genes" and not EyeGEx genes, as when I first read this, I thought the authors were referring to genes with a significant eQTL (eGenes). In the Results section on lines 135-137, I would add the word "compared to GTEx tissues, as follows: " these were significantly enriched for genes with a highly specific expression in the retina from the EyeGEx database compared to GTEx tissues (44/242 retina-enriched genes, p=0.000273)"

Minor comments:

* If the authors want to use subheaders in the abstract, I would split the Results section into Methods and Results, and add a bit more information in the Methods section.
* In the abstract, it might be informative to add the ration between retina and RPE-specific chromatin interactions.
* In the following sentence in the abstract: "We zoomed in on tissue-specific chromatin interactions at the ABCA4 locus using high-resolution UMI-4C assays. I might change the words "tissue-specific" to "retina- or RPE-specific".
* In the Introduction I would edit the sentence on lines 94-96, as follows: "We therefore generated genome-wide chromatin interaction maps by applying in situ Hi-C9 and H3K4me3 HiChIP24 to neural retina and RPE/choroid from human adult post-mortem donor eyes and performed a comparative 3D genome analysis between the two retinal tissues."
* In Results on page 7, lines 150-152, I would put "(Fig 1g)" after the first half of the sentence, as follows: "Gene Ontology enrichment analysis also indicated an involvement of genes associated with the visual system in (differential) chromatin looping in the neural retina (Fig 1g),"
* On page 8, lines 156-158, the authors might want to state what are the most abundant cell types in retina and RPE that were enriched for expression of genes at differential chromatin loops, e.g. photoreceptors for retina, and RPE and immune cells for RPE/choroid.
* On page 10, lines 213-215, it is not precise that the EyeGEx data is part of the GTEx dataset. The EyeGEx expression files are posted on the GTEx portal, but it is a separate study (Ratnapriya et al, 2019). Furthermore, while the retinal tissues from Ratnapriya et al, 2019 was indeed derived from neural retina, it does appear to contain some contamination of RPE in the samples based on expression of RPE markers such as RPE65. I would change the end of the sentence on lines 214-215 to: "while the retinal tissue from the EyeGEx database primarily contain neural retina."
* Can the authors add a reference to the first part of the sentence on page 10, lines 227-228: "As single-cell RNA sequencing experiments have indicated that many known IRD genes are expressed in a cell-type specific manner".
* On page 13, lines 295-296, can the authors add in parentheses how much KB upstream ABCD3 is of ABCA4: "UMI-4C interaction profiles confirmed extended interactions in the neural retina, as far upstream as the ABCD3 gene (XXXkb)"
* In the Methods, under section "Tissue preparation and nuclei isolation", what is the postmortem interval of the retina samples used in the study? Is it expected to affect the 3D confirmation?
* In the Methods section, can the authors add the GTEx version used on lines 550-552: "Tissue-specific expression of genes in differential windows or at differential loops was evaluated using the GTEx dataset".
* In the Methods section starting on line 579 "Integration of bulk and single-cell transcriptomic and epigenomic datasets from human donor retina" please clarify the Single-nucleus RNA-seq for retina study used in the analysis in this paper.

**Reviewer 2**

The stated goal of this manuscript by D'haene and colleagues is "To understand the role of differential chromatin architecture in establishing tissue-specific expression patterns at IRD loci in the human neural retinal and the RPE". To achieve this, they pursued complementary approaches including Hi-C and HiChiP to map and compare chromatin interactions in adult human retina and RPE with an emphasis on inherited retinal disease gene loci. The authors then focused on the ABCA4 disease-gene locus to further refine their understanding of RPE and retinal cis-regulatory elements and performed UMI-4C, data mining, and in vivo reporter assays to support their findings and the predictive value of their chromatin conformation assays.

The major strengths of this work include the utility of the newly generated datasets as powerful resources for the field, the comparison between different experimental techniques and different tissues, and the focus on disease loci. This work further helps to clarify a field that initially stated that 3D chromatin interactions were largely conserved across tissues and cell lines. Weaknesses include 1) concern that the manuscript may be overly focused on differences between RPE and retina, when in many cases it seems that the similarities are more common than the differences; 2) concern that some of the differences are technical in nature (potentially from low coverage of RPE). In sum, the strengths outweigh the weaknesses and I believe that this manuscript will be of special interest to others in the field.

I believe the manuscript would be further improved by addressing the following questions and concerns:

1) The single most important deliverable from these studies would be a table of all the putative enhancer-promoter pairs supported by these assays in retina and in RPE (not just the differential ones). That would be an extremely useful resource for researchers in this field. Is that available in an easily searchable format? If yes, it should be emphasized and made more clearly available. If no, providing that resource would significantly increase the impact of this work.

2) Despite stating in the methods that this information is available, this reviewer cannot find information in Table S5 regarding postmortem interval of donor tissue or cause of death.

3) It should be stated more clearly in the manuscript and the methods that although n=4 for distinct tissues used in Hi-C and n=2 for HiChIP, some of those tissues are from the same donors and therefore are not fully independent biological replicates.

4) Since tissue was used from both female and male donors, it would be useful to know if any sex specific differences were observed and whether the design is sufficiently powered to detect sex specific differences including chromatin architecture on the Y chromosome.

5) Further explanation would be helpful for why 6,884 loops were identified in retina by Hi-C, while only 2,902 loops were found in RPE and what the consequences of that skew are on downstream analysis and interpretation. Is this because a RPE is much more heterogeneous with respect to cell type than retina, because chromatin is organized in a significantly different way in cells of the RPE vs. retina, or does this reflect poorer overall coverage for RPE? Please provide evidence that the coverages are sufficiently balanced to make conclusions about tissue-specific patterns of chromatin conformation.

6) This reviewer is unclear what analysis was performed to arrive at Sup Fig S9d.

**Authors’ response to reviewers**

Response to reviewer reports


Reviewer #1:
---------------

The manuscript "Comparative 3D genome analysis between neural retina and RPE reveals differential cis-regulatory interactions at retinal disease loci" by D'haene et al., compares for the first time genome-wide chromatin 3D interactions between human neural retina and retinal pigment epithelium (RPE)/choroid (vascular bed in outer layer of retina) using Hi-C and a higher resolution assay, HiChIP. They then investigate how the retina-specific and RPE-specific interaction maps can help identify cis-regulatory effects on 290 inherited retinal degeneration (IRD) disease genes, and find differential chromatin interactions are enriched in 60% of IRD genes. They further describe the biological processes enriched for genes in retina or RPE-specific chromatin contacts. Finally, the authors focus on an IRD gene example, ABCA4 that makes up a significant fraction of early onset retinal degeneration cases and is the most common autosomal recessive IRD gene expressed in both neural retina and RPE. They identify tissue-specific chromatin interactions in ABCA4 in neural retina and RPE, and use a targeted high resolution assay (UMI-4C), in vivo enhancer assays in zebrafish, and integration with single cell epigenomic data to provide additional support and further characterize the tissue-specific cis regulatory elements in ABCA4. The analyses presented in this paper appropriately support the conclusions of this study.

This work provides an important resource for the study of both rare and common retina-related eye disease genes and variants, and proposes an experimental and computational workflow that can be applied to other tissue and disease systems. The manuscript is well written. I have a few suggestions and mostly minor comments I recommend addressing:

>> We thank the reviewer for the overall appreciation of our manuscript.

Major comments:

1) If there are enough IRD genes known to specifically affect the RPE compared to neural retina, it would be interesting to evaluate the concordance of RPE-specific IRD genes with genes enriched in RPE-specific chromatin loops or chromatin interaction with promoters, and IRD genes manifested specifically in the neural retina and the retina-specific chromatin interactions.

>> We thought it was an excellent suggestion by the reviewer to assess whether RPE/choroid or neural retina specific IRD genes might be more strongly associated with loops specific to either tissue. Using the single-cell RNA-seq dataset generated by Cowan et al. 2020 (PMID: 32946783), we scaled gene expression across all identified cell types (Z-score normalization). Genes with cell-type specific expression in the RPE/choroid where then identified by filtering for genes with a Z-score > 2 in at least one cell-type found in the RPE/choroid layer ("RPE", "PER", "FB_01", "FB_02", "FB_03", "END_01", "END_02", "END_03", "CM", "NK", "TCell", "MO_01", "MO_02", "MO_03", "MAST"). Similarly, to identify genes with cell-type specific expression in the neural retina, we filtered for genes with a Z-score > 2 in at least one cell-type found in the neural retina (all other cell-types excluding the ones mentioned above). This resulted in a set of 74/290 IRD genes with cell-type specific expression in the RPE/choroid and 239/290 IRD genes with specific expression in at least one cell type of the neural retina (43 of these genes showed specific expression in cell types of both the neural retina and the RPE/choroid).

Also, we now used these sets of “retina-specific” and “RPE/choroid-specific” IRD genes to evaluate their enrichment at retina- or RPE/choroid-specific loops as the reviewer suggested. And indeed, at retina-specific Hi-C and HiChIP loops we found a highly significant enrichment of retina-specific IRD genes (2.3-fold and 1.5-fold respectively for differential Hi-C and HiChIP loops), while RPE/choroid specific IRD genes were not enriched at these retina-specific loops (Fig 1f). At Hi-C loops identified in the RPE/choroid and RPE/choroid-specific HiChIP loops, we observed a 1.7-fold and 1.3-fold enrichment of RPE/choroid-specific IRD genes respectively, although this was not significant due to the smaller numbers of RPE/choroid loops and therefore smaller gene sets (Fig 1f). These results further indicate that tissue-specific chromatin interactions are likely involved in tissue-specific expression of IRD genes. These new analyses have been added to the text and figures in the results section as follows:

“Next, we evaluated whether IRD genes with specific expression in the neural retina or RPE/choroid would be more strongly associated with tissue-specific loops. Using scRNA-seq data from adult human retina3 and by scaling gene expression across all identified cell types, 74/290 IRD genes were identified as having enriched expression in at least one cell type within the RPE/choroid (Z-score > 2), while 239/290 IRD genes showed enriched expression in at least one cell type of the neural retina (Z-score > 2)(Sup Fig S5, Table S1). We found that retina-specific IRD genes were strongly enriched at (differential) Hi-C loops in the neural retina, while RPE/choroid-specific IRD genes were not enriched at these retinal loops (Fig 1f). Similarly, at Hi-C loops identified in the RPE/choroid, we observed a 1.7-fold enrichment of only RPE/choroid-specific IRD genes, although this was not significant due to the small number of loops and therefore small gene sets (Fig 1f).”

“Moreover, we again found a stronger enrichment when only considering IRD genes with specific expression in cell types of the neural retina (119/208 retina-specific IRD genes at retinal HiChIP loops, p=2.042e-07), while RPE/choroid-specific IRD genes were not enriched at retina-specific HiChIP loops (Fig 2c). Conversely, only RPE/choroid-specific IRD genes were slightly enriched (1.3-fold) at RPE/choroid-specific HiChIP loops (not significant), while we also observed a significant depletion of retina-enriched genes from the EyeGEx database (p=0.0046) (Fig 2c).”

Also, the information on the specific expression of IRD genes has been added to Table S1, which now gives an overview for all IRD genes of (1) their association with differential chromatin interactions, (2) their specific expression in cell types of the neural retina and/or RPE/choroid and (3) their interaction (either stable, retina- or RPE/choroid-specific) with previously identified cCREs with activity in the neural retina or RPE. In our opinion, by providing an easily searchable overview of all this information in one place, Table S1 has become a valuable tool for further research.


2) How do the retina loops found in this study compare to those found in Marchal et al., 2022?

>> This is again a great suggestion by the reviewer and a crucial analysis to add to the manuscript. We have now assessed the overlap with the set of retinal Hi-C loops reported by Marchal et al. Despite differences in experimental protocol, data resolution and analytical tools, we found a high degree of overlap with both the Hi-C (60%) and HiChIP loops (75%, after subsetting Hi-C loops for overlap with invariant H3K4me3 at TSS) we identified in the neural retina. This is an important validation of the datasets included in our work and thus we have added this to the results section and figures as follows:

“60% of neural retina loops (4081/6884) correspond to loops previously identified in the same tissue by Marchal et al.”

“…, while 75% of retinal Hi-C loops previously identified by Marchal et al. correspond to neural retina HiChIP loops (Sup Fig S9d).”

3) The authors define retina-enriched genes from the EyeGEx database (Ratnapriya et al , Nature Genetics 2019) as genes with >10-fold higher expression in retina compared to >=42 of 53 GTEx (v7) tissues. It would be helpful to define what is meant by retina-enriched genes in the Results section, i.e., genes expressed at higher levels in retina compared to GTEx tissues. The authors might want to consider calling these genes "retina-specific genes" and not EyeGEx genes, as when I first read this, I thought the authors were referring to genes with a significant eQTL (eGenes). In the Results section on lines 135-137, I would add the word "compared to GTEx tissues, as follows: " these were significantly enriched for genes with a highly specific expression in the retina from the EyeGEx database compared to GTEx tissues (44/242 retina-enriched genes, p=0.000273)"

>> We do agree that the term “EyeGEx genes” could be misleading. The text now clearly refers to these genes as having a specific expression in the retina. The exact method that was used within the EyeGEx paper to arrive at this list of retina-enriched genes is mentioned in the Methods section.

Minor comments:

* If the authors want to use subheaders in the abstract, I would split the Results section into Methods and Results, and add a bit more information in the Methods section.

>> Genome Biology guidelines require the inclusion of these specific sections to the abstract. We are therefore constrained by these formatting rules. In addition, there is a Word limit of 250, not allowing extensions.

For guidelines see: https://genomebiology.biomedcentral.com/submission-guidelines/preparing-your-manuscript/research

“The abstract must include the following separate sections:
• Background: the context and purpose of the study
• Results: the main findings
• Conclusions: a brief summary and potential implications”


* In the abstract, it might be informative to add the ratio between retina and RPE-specific chromatin interactions.

>> Thank you for the suggestion. We thought it might be even more informative to add how many interactions with previously identified candidate CREs were identified using our data in both tissues. Hence, the following sentence has been added to the abstract:

“We observed chromatin looping between active promoters and 32,425 and 8,060 candidate CREs (cCREs) in the neural retina and RPE/choroid respectively.”


* In the following sentence in the abstract: "We zoomed in on tissue-specific chromatin interactions at the ABCA4 locus using high-resolution UMI-4C assays. I might change the words "tissue-specific" to "retina- or RPE-specific".

>> This has been adapted in the abstract.

* In the Introduction I would edit the sentence on lines 94-96, as follows: "We therefore generated genome-wide chromatin interaction maps by applying in situ Hi-C9 and H3K4me3 HiChIP24 to neural retina and RPE/choroid from human adult post-mortem donor eyes and performed a comparative 3D genome analysis between the two retinal tissues."

>> We agree that this phrasing reflects the experimental design of our study more clearly (i.e. doing Hi-C and HiChIP on both retinal tissues separately). We therefore adopted this suggestion in the introduction.

* In Results on page 7, lines 150-152, I would put "(Fig 1g)" after the first half of the sentence, as follows: "Gene Ontology enrichment analysis also indicated an involvement of genes associated with the visual system in (differential) chromatin looping in the neural retina (Fig 1g),"

>> This has been adapted.

* On page 8, lines 156-158, the authors might want to state what are the most abundant cell types in retina and RPE that were enriched for expression of genes at differential chromatin loops, e.g. photoreceptors for retina, and RPE and immune cells for RPE/choroid.

>> We incorporated this suggestion in the text, which now reads: “… subsets of genes at differential chromatin loops, displayed specific expression in the most abundant cell types of either the neural retina (photoreceptors) or the RPE/choroid (RPE, fibroblasts, endothelial and immune cells) (Fig 1h & Sup Fig S7-8).”

* On page 10, lines 213-215, it is not precise that the EyeGEx data is part of the GTEx dataset. The EyeGEx expression files are posted on the GTEx portal, but it is a separate study (Ratnapriya et al, 2019). Furthermore, while the retinal tissues from Ratnapriya et al, 2019 was indeed derived from neural retina, it does appear to contain some contamination of RPE in the samples based on expression of RPE markers such as RPE65. I would change the end of the sentence on lines 214-215 to: "while the retinal tissue from the EyeGEx database primarily contain neural retina."

>> We agree that this was indeed unclear and have changed this sentence to: “…, while the retinal tissue from the EyeGEx database (integrated within the GTEx dataset) primarily contains neural retina30.”

* Can the authors add a reference to the first part of the sentence on page 10, lines 227-228: "As single-cell RNA sequencing experiments have indicated that many known IRD genes are expressed in a cell-type specific manner".

>> A reference to the article by Cowan et al. (2020) has been added.

* On page 13, lines 295-296, can the authors add in parentheses how much KB upstream ABCD3 is of ABCA4: "UMI-4C interaction profiles confirmed extended interactions in the neural retina, as far upstream as the ABCD3 gene (XXXkb)"

>> We have now specified this in the text: “…as far upstream as the ABCD3 gene (~300 kb),...”

* In the Methods, under section "Tissue preparation and nuclei isolation", what is the postmortem interval of the retina samples used in the study? Is it expected to affect the 3D confirmation?

>> We have added the post-mortem intervals of included donors to Table S5.

Although it has been documented that longer post-mortem intervals can affect RNA integrity and therefore transcriptome studies (Ferreira et al., 2018, PMID: 29440659; Cowan et al., 2020, PMID: 32946783), DNA is known to be relatively stable over long post-mortem periods. The association of nucleosomal DNA with core histones (Huang et al., 2006, PMID: 16574239), as well as DNA methylation and histone methylation and acetylation have been shown to be generally stable for post-mortem intervals <24h (Jarmasz et al., 2019, PMID: 30635019). Based on these findings, we therefore assume that 3D chromatin interactions would also be stable for the post-mortem intervals reported for the donors included in this study (ranging from 3-18h). Although it must be noted that the impact of post-mortem interval length on 3D chromatin changes has not been assessed in detail.

* In the Methods section, can the authors add the GTEx version used on lines 550-552: "Tissue-specific expression of genes in differential windows or at differential loops was evaluated using the GTEx dataset".

>> The GTEx version (v8) has now been specified in the text.

* In the Methods section starting on line 579 "Integration of bulk and single-cell transcriptomic and epigenomic datasets from human donor retina" please clarify the Single-nucleus RNA-seq for retina study used in the analysis in this paper.

>> The single-nucleus RNA-seq data was obtained from the same publication as the single-nucleus ATAC-seq data described in the previous sentences. However, a reference specifically in this sentence was indeed missing and has been added to the text.


 
Reviewer #2:
---------------

The stated goal of this manuscript by D'haene and colleagues is "To understand the role of differential chromatin architecture in establishing tissue-specific expression patterns at IRD loci in the human neural retinal and the RPE". To achieve this, they pursued complementary approaches including Hi-C and HiChiP to map and compare chromatin interactions in adult human retina and RPE with an emphasis on inherited retinal disease gene loci. The authors then focused on the ABCA4 disease-gene locus to further refine their understanding of RPE and retinal cis-regulatory elements and performed UMI-4C, data mining, and in vivo reporter assays to support their findings and the predictive value of their chromatin conformation assays.

The major strengths of this work include the utility of the newly generated datasets as powerful resources for the field, the comparison between different experimental techniques and different tissues, and the focus on disease loci. This work further helps to clarify a field that initially stated that 3D chromatin interactions were largely conserved across tissues and cell lines. Weaknesses include 1) concern that the manuscript may be overly focused on differences between RPE and retina, when in many cases it seems that the similarities are more common than the differences; 2) concern that some of the differences are technical in nature (potentially from low coverage of RPE). In sum, the strengths outweigh the weaknesses and I believe that this manuscript will be of special interest to others in the field.

>> We thank the reviewer for the overall appreciation of our manuscript and for pointing out strengths as well as limitations and suggestions for improvement and for increasing the impact of our work.

I believe the manuscript would be further improved by addressing the following questions and concerns:

1) The single most important deliverable from these studies would be a table of all the putative enhancer-promoter pairs supported by these assays in retina and in RPE (not just the differential ones). That would be an extremely useful resource for researchers in this field. Is that available in an easily searchable format? If yes, it should be emphasized and made more clearly available. If no, providing that resource would significantly increase the impact of this work.

>> We agree that providing this information would greatly increase the usability and searchability of our data for other researchers. We have therefore annotated all stable and retina- and RPE-specific promoter loops identified through HiChIP analyses with previously identified candidate cis-regulatory elements (cCREs) active in neural retina or RPE (Cherry et al., 2020). Specifically, using HiChIP stable, retina-specific and RPE-specific loops, we identified 134,374 neural retina loops (stable & retina-specific) connecting 15,819 TSSs to 32,425 retinal cCREs; and 118,461 loops in RPE/choroid (stable & RPE/choroid-specific) connecting 13,190 TSSs to 8,060 RPE cCREs. The full set of loops with cCRE annotation has now been provided as a supplemental data file (Sup Data 8).

We have added a sentence to the abstract and a paragraph to the HiChIP section in the results to highlight this analysis:

“We observed chromatin looping between active promoters and 32,425 retinal candidate CREs (cCREs) and 8,060 RPE cCREs in the neural retina and RPE/choroid respectively.”

“Next, we used these stable and retina-/RPE-specific loops to identify interactions between promoters and candidate cis-regulatory elements (cCREs) with activity in the retina or RPE previously identified by Cherry et al. (2020) (Sup Data 8). Specifically, using HiChIP stable, retina-specific and RPE-specific loops, we identified 134,374 neural retina loops (stable & retina-specific) connecting 15,819 TSSs to 32,425 retinal cCREs; and 118,461 loops in RPE/choroid (stable & RPE/choroid-specific) connecting 13,190 TSSs to 8,060 RPE cCREs.”

Also, to improve usability of our data for other researchers we have now made an overview of promoter-cCRE associations (based on HiChIP loops) for all known IRD genes in Table S1. As this table now also contains information on specific expression of IRD genes in cell types of either the neural retina or RPE/choroid (as requested by reviewer #1), expression of these genes can now be directly linked to their interaction with cCREs active in these specific tissues.

2) Despite stating in the methods that this information is available, this reviewer cannot find information in Table S5 regarding postmortem interval of donor tissue or cause of death.

>> This information has been added to Table S5.

3) It should be stated more clearly in the manuscript and the methods that although n=4 for distinct tissues used in Hi-C and n=2 for HiChIP, some of those tissues are from the same donors and therefore are not fully independent biological replicates.

>> Thank you for this remark. We agree that this is important information for readers of the manuscript. To be fully transparent, this has now been specified in both the results and methods sections as follows:

In the Results section:

“We used in situ Hi-C on post-mortem human donor retina to map 3D genomic interactions in the adult neural retina (n=4, four eyes from three donors), as well as the RPE/choroid layer (n=4, four eyes from three donors) (Fig 1a).”

“To identify cis-regulatory interactions involving active promoters at a higher resolution and with greater sensitivity, we performed HiChIP24 for H3K4me3 in both human adult neural retina (n=2, two eyes from one donor) and RPE/choroid (n=2, two eyes from two donors).”

In the Methods section:

“Crosslinked nuclei from four neural retina and four RPE/choroid samples (derived from four eyes obtained from three donors) were used to construct Hi-C libraries, …”

“HiChIP was performed as previously described24 using cross-linked nuclei from two neural retina and RPE/choroid samples (derived from two eyes, obtained from one and two donors respectively) (Table S5).”

4) Since tissue was used from both female and male donors, it would be useful to know if any sex specific differences were observed and whether the design is sufficiently powered to detect sex specific differences including chromatin architecture on the Y chromosome.

>> Our study was not sufficiently powered to detect sex specific differences. Although both female and male samples were included, the distribution of these samples across the different assays (UMI-4C, Hi-C, HiChIP) was not equal. For example, HiChIP assays were only performed on female samples. Hi-C interactions matrices also displayed lower quality for the Y chromosome since this was only derived from a limited number of samples (one male sample for the neural retina, two male samples from the same individual for the RPE/choroid). The information on the numbers of male and female samples included in all assays can be found in Table S5.


5) Further explanation would be helpful for why 6,884 loops were identified in retina by Hi-C, while only 2,902 loops were found in RPE and what the consequences of that skew are on downstream analysis and interpretation. Is this because a RPE is much more heterogeneous with respect to cell type than retina, because chromatin is organized in a significantly different way in cells of the RPE vs. retina, or does this reflect poorer overall coverage for RPE? Please provide evidence that the coverages are sufficiently balanced to make conclusions about tissue-specific patterns of chromatin conformation.

>> We agree with the reviewer that this is a striking observation that deserved more attention in the manuscript. First, we can provide evidence that the coverages are sufficiently balanced. The total read coverage, which has now been added to the Methods section, was similar for neural retina and RPE/choroid samples (1.8 billion reads for neural retina, 2.1 billion reads for RPE/choroid). This is also reflected by similar numbers of contacts that are present within the merged contact matrices (1.13 billion and 1.34 billion for neural retina and RPE/choroid respectively, numbers were already mentioned in the results section). The number of TAD boundaries we identified in both tissues were comparable (3,905 and 3,785 boundaries in neural retina and RPE/choroid respectively, also mentioned in the results section), yet the number of loops is indeed unbalanced indicating less focal/punctate interactions were found in the RPE/choroid than in the neural retina interaction matrix.

We can speculate that this is the result of the cell type composition of both tissues. While the neural retina samples are mainly composed of photoreceptors (~80%) and the interaction matrices therefore reflect an almost cell-type specific signal, the RPE/choroid samples are very heterogeneous (being composed of RPE, fibroblasts, endothelial and immune cells). As a result, the punctate signal from cell-type specific loops will be diluted in the RPE/choroid interaction maps. An alternative explanation may come from a lower degree of cis-regulatory complexity in the RPE/choroid compartment versus neural retina, given that neurons in general are highly complex cell types from a regulatory point of view (Closser et al., 2022, PMID: PMID: 34727520).

This type of imbalance has also been observed by others when for example when assessing open chromatin regions in both neural retina and RPE/choroid (Cherry et al., 2020), with the neural retina exhibiting almost twice as many peaks as the RPE/choroid (21,368 vs. 12,212 open chromatin regions).

We have added an extra sentence to the discussion to further emphasize this topic. The section now reads:

“Yet, it is important to note that even chromatin interaction mapping at the tissue level foregoes the underlying cellular complexity, as the resulting interaction maps reflect contact frequencies derived from a mixture of different cell types. In this case, we observed that interaction data from neural retina primarily reflects contacts derived from the most abundant cell types by far, namely the photoreceptors. This was clearly exemplified by the photoreceptor-specific expression of most genes near differential contacts gained in the neural retina maps. The RPE/choroid layer, on the other hand, is comprised of a mixture of epithelial, endothelial, fibroblast and immune cells and the resulting interaction maps are therefore expected to reflect an average contact frequency across these different cell types. This might also explain the imbalance we observed in the number of chromatin loops that could be identified in neural retina vs. RPE/choroid Hi-C matrices. Despite similar sequencing coverage and contact numbers, more than twice as many Hi-C loops were identified in the neural retina. We speculate that the punctate signal from cell-type specific loops might be diluted in the RPE/choroid interaction maps due to its heterogeneous composition. An alternative explanation though may come from a lower degree of cis-regulatory complexity in the RPE/choroid compartment versus neural retina, given that neurons in general are highly complex cell types from a regulatory point of view37. Future interaction mapping at the cell-type level will be required to disentangle this complexity.”

6) This reviewer is unclear what analysis was performed to arrive at Sup Fig S9d.

>> We realize this might not have been sufficiently clear from the text and the caption and thank the reviewer for pointing this out. To compare Hi-C loops with HiChIP loops and perform an overlap between them, we filtered the list of Hi-C loops to only retain those that could be found at an active TSS, displaying invariant H3K4me3 in neural retina and RPE/choroid samples, as all HiChIP loops used for the FitHiChIP analysis fulfilled these criteria as well. Specifically, we used FitHiChIP to provide H3K4me3 annotation for the Hi-C loops. Sup Fig S9d represents the fraction of the filtered Hi-C loops that were also identified through HiChIP analyses.

The approach we used for this analysis has now been detailed in the Methods section as follows:

“To determine the overlap between Hi-C loops and HiChIP loops identified in both retinal tissues, FitHiChIP29 was used to annotate Hi-C loops with H3K4me3 at 5 kb resolution. Hi-C loops were then filtered to only retain those loops with characteristics that resemble those of HiChIP loops included in the differential FitHiChIP analysis, i.e. category ND-ND and 2 kb up- or downstream from a transcription start site. Subsequently, we performed an overlap between (1) filtered retinal Hi-C loops and stable or retina-specific HiChIP loops and (2) filtered RPE Hi-C loops and stable or RPE-specific HiChIP loops. The same approach was used to perform the overlap between retinal Hi-C loops identified by Marchal et al. (2022)12 and the set of stable or retina-specific HiChIP loops.”

**Second round of review**

**Reviewer 1**

The authors did a good job in addressing my comments and those of the second reviewer. I only have a few minor edits following the revisions:

1. I would add the word ‘(Methods)’ after the sentence “and by scaling gene expression across all identified cell types,” on line 157, to point the reader to how the gene expression scaling across cell types was done.
2. Can the authors specify what statistical test was used in the legend of Fig. 1f, and in a few other locations in the Results section where a p-value is specified but not the statistical test applied, e.g. for gene expression enrichment or comparative analysis. The authors can point to the Methods section if the statistical test is described there. For example, this is missing in the following sentence on lines 160-162: “We found that retina-specific IRD genes were strongly enriched at (differential) Hi-C loops in the neural retina (YYY test, p=XXX), while RPE/choroid-specific IRD genes were not enriched at these retinal loops (Fig 1f; YYY test, p=XXX).” Or on lines 139-140: “(44/242 retina-enriched genes from the EyeGEx database compared to other GTEx tissues, p=0.000273)”. Can the authors add in the parentheses what statistical test was applied to get the p-value?
3. In the abstract, on line 28, the authors should add a comma before the word “respectively”, and also throughout the paper where “respectively” is used.
4. I would remove the words “(integrated within the GTEx dataset)” on line 224, as this is not exactly the case. The EyeGEx is a separate dataset from the GTEx dataset and was removed from the GTEx portal.
5. I would recommend the authors add the postmortem interval range after “post-mortem circulation time,” in the Methods section on line 483.
